# Endovascular treatment for young patients with acute large vessel occlusion stroke in China: analysis of the ANGEL-ACT registry

**DOI:** 10.3389/fneur.2023.1255043

**Published:** 2023-10-20

**Authors:** Bin Han, Raynald, Dapeng Sun, Xu Tong, Baixue Jia, Anxin Wang, Dapeng Mo, Feng Gao, Ning Ma, Thanh N. Nguyen, Zhongrong Miao

**Affiliations:** ^1^Shanxi Key Laboratory of Brain Disease Control, Department of Neurology, Shanxi Provincial People's Hospital, Taiyuan, China; ^2^Department of Interventional Neuroradiology, Beijing Tiantan Hospital, Capital Medical University, Beijing, China; ^3^Department of Neurology, Beijing Tiantan Hospital, Capital Medical University, Beijing, China; ^4^China National Clinical Research Center for Neurological Diseases, Beijing Tiantan Hospital, Capital Medical University, Beijing, China; ^5^Department of Neurology, Radiology, Boston Medical Center, Boston, MA, United States

**Keywords:** thrombectomy, endovascular treatment, large vessel occlusion, acute ischemic stroke, young patients, outcomes

## Abstract

**Background:**

The incidence of acute ischemic stroke caused by large vessel occlusion is relatively infrequent in the young adult population. We sought to evaluate their clinical outcomes after endovascular treatment (EVT) and stroke etiology compared with older patients.

**Methods:**

We examined data from the ANGEL-ACT registry, a nationwide study in China focusing on EVT for acute ischemic stroke. We compared two age groups: <50 years old and ≥50 years old. Our analysis focused on outcome measures such as the 90-day modified Rankin Scale (mRS) score, mortality, and symptomatic intracranial hemorrhage (sICH). We adjusted for confounding variables.

**Results:**

We included 1,691 patients, and 216 patients (13%) were <50 years old. Young patients had lower median National Institutes of Health Stroke Scale (NIHSS) scores (14 vs. 17, *P* < 0.001) and fewer cardiovascular comorbidities than older patients. Underlying intracranial atherosclerosis disease (ICAD) was higher in young patients (39.4 vs. 28.7%, *P* = 0.001). Clinical outcome was less favorable in older compared to younger patients (mRS shift: 0.76 [95% confidence interval (CI), 0.58–0.99]); functional independence [mRS score 0–2] 61% vs. 39% (adjusted odds ratio (OR), 0.7 [95% CI, 0.51–0.97]). Mortality and sICH did not differ between groups. Onset to puncture time (OTP) was longer in young patients (357 min vs. 294 min, *P* = 0.001).

**Conclusion:**

An estimated 13% of patients who underwent endovascular thrombectomy for acute ischemic stroke were <50 years old. Symptomatic underlying ICAD was more prevalent in the younger patient population. Despite a prehospital delay, younger patients exhibited more favorable outcomes than their older counterparts.

## Introduction

Endovascular treatment (EVT) has become the established therapy for patients experiencing acute ischemic stroke in the anterior circulation caused by intracranial large vessel occlusion (LVO) (1, 2). Notably, the majority of patients undergoing EVT are elderly individuals, which is consistent with the general incidence of stroke. Conversely, LVO-related stroke is relatively rare among younger patients (<50 years) (3), resulting in limited data on outcomes following EVT and underlying causes in this population. In a pooled patient-level meta-analysis of the highly effective reperfusion evaluated in the Multiple Endovascular Stroke Trials collaboration, a favorable outcome was observed in 56% of patients aged 18 to 49 in the intervention group, compared to 47% in the control group (1). Though the treatment effect of EVT in this subgroup did not reach statistical significance, the trend favored EVT. Consequently, further investigation of the efficacy of EVT in this age group is of importance.

The significance of stroke in younger patients is immense from a public health perspective, as it may lead to a higher lifetime disability burden and potentially catastrophic consequences for individuals in this working age group (4, 5). Most studies focusing on LVO-related stroke in younger patients had limited sample sizes, were single-center in nature, or did not specifically evaluate the impact of EVT (6–10). In this context, the primary objective of the current investigation was to compare the clinical outcomes following EVT between young and older patients. The secondary aim was to evaluate the stroke etiology in young patients with LVO stroke who underwent EVT in a large nationwide registry.

## Methods

The data that support the findings of this study are available from the corresponding authors. This manuscript follows the Strengthening the Reporting of Observational Studies in Epidemiology (STROBE) reporting guideline (11).

### Study population

The data utilized in this study were obtained from the ANGEL-ACT registry, a prospective nationwide registry consisting of 1,793 consecutive patients who underwent EVT for acute LVO at 111 hospitals from 26 provinces in China between November 2017 and March 2019 (12). The registry enrolled patients who met the following inclusion criteria: (1) age ≥18 years; (2) diagnosed with acute ischemic stroke caused by imaging-confirmed intracranial LVO, including the internal carotid artery, middle cerebral artery (M1/M2), anterior cerebral artery (A1/A2), vertebral artery, basilar artery, and posterior cerebral artery (P1); and (3) initiated any form of EVT, including mechanical thrombectomy, intra-arterial thrombolysis, balloon angioplasty, stenting, and other mechanical fragmentation. The ANGEL-ACT registry aimed to provide insight into the current status of EVT in real-world clinical practice for patients with acute LVO in China. The protocol was approved by the ethics committees of Beijing Tiantan Hospital and each participating site. Each participant or their representative provided written informed consent before enrollment in the study.

### Data collection

The registry collected various data, including demographic characteristics, vascular risk factors, vital signs, stroke severity (measured by the National Institutes of Health Stroke Scale [NIHSS]), laboratory tests, neurovascular images, endovascular procedural details, peri-procedural management and complications, functional outcome (measured by the modified Rankin Scale [mRS]), and adverse events within 90 days after the procedure. The diagnostic workup of stroke etiology was according to the local site protocol and/or national guidelines (13). The stroke subtype was defined according to the TOAST criteria (14). Only investigators trained and qualified to examine the NIHSS and mRS recorded the scores. An imaging core laboratory blinded to the clinical data and outcomes evaluated baseline computed tomography (CT)/magnetic resonance (MR), CT angiography (CTA)/MR angiography (MRA), digital subtraction angiography (DSA), and post-procedural CT. Follow-up CTs were performed immediately and 24 ± 2 h after the procedure, with an additional CT obtained at any time of neurological deterioration. Two neuroradiologists independently assessed all images, with a third available for adjudication when needed. Reconstructed images obtained from pre-procedural scans such as computed tomography angiography (CTA) or magnetic resonance angiography (MRA) were validated through intraprocedural digital subtraction angiography (DSA) to establish the classification. The core laboratory image interpretation included early ischemic changes using the Alberta Stroke Program Early CT Score (ASPECTS) for anterior circulation stroke and posterior circulation ASPECTS (pc-ASPECTS) for posterior circulation stroke, occlusion site, tandem lesions, symptomatic underlying intracranial atherosclerotic disease (ICAD, defined as fixed stenosis degree >70% or >50% with distal blood flow impairment or evidence of repeated re-occlusion), baseline and post-procedural modified thrombolysis in cerebral infarction (mTICI), and occurrence of intracranial hemorrhage (ICH) on post-treatment imaging. For patients whose images could not be obtained, site-reported data were used. The imaging review criteria of local investigators were the same as those of the core laboratory, except that ICAD could also be determined according to prior images indicating a stenotic lesion at the occlusion site.

### Outcome measurement

The primary outcome measure of this study was the mRS score at 90 days. Trained investigators, who were blinded to the baseline information, conducted telephone interviews based on a standardized interview protocol to assess the 90-day mRS score.

Secondary outcome measures included the proportion of patients with mRS scores of 0–1, 0–2, and 0–3 at 90 days, changes in the National Institutes of Health Stroke Scale (NIHSS) score at 24 h and 7 days as assessed by local neurologists, successful recanalization defined as a mTICI score of 2b to 3, the time intervals from door-to-puncture and puncture-to-recanalization, and the number of passes required for thrombectomy.

Safety outcomes were also assessed, including death within 90 days, any intracranial hemorrhage (ICH) and symptomatic ICH within 24 h based on the Heidelberg Bleeding Classification, and procedure-related complications such as intraprocedural embolization, arterial perforation, arterial dissection, and vasospasm requiring treatment.

### Statistical analysis

All data were reported as median (interquartile range [IQR]) for continuous and ordinal variables, and as a number (percentage) for categorical variables. The baseline characteristics of the two groups were compared using the Mann–Whitney *U*-test for continuous and ordinal variables, and Pearson's chi-square test or Fisher's exact test for categorical variables. For the outcome measures, adjusted odds ratios (ORs), common OR, or β-coefficients with their 95% confidence intervals (CIs) were calculated using a binary or ordinal logistic regression model, or generalized linear model as appropriate. Baseline variables with a significant difference of *p* < 0.05 were considered potential confounders and adjusted accordingly. Additionally, a propensity score was developed for adjustment, which was derived from a logistic regression model that included all baseline variables, including age. To explore treatment effect modification on the primary outcome, subgroup analyses were performed based on sex, prior intravenous thrombolysis, stroke subtype by Trial of ORG 10,172 in Acute Stroke Treatment (TOAST) criteria, occlusion circulation, underlying ICAD, and tandem lesions. Treatment effect size heterogeneity across the subgroups was assessed by including the corresponding multiplicative interaction term in the logistic regression model with propensity score adjustment. All analyses were conducted using SAS software version 9.4 (SAS Institute Inc, Cary, NC). A two-sided *p*-value of < 0.05 was considered statistically significant.

## Results

The ANGEL-ACT Registry included 1,793 patients, of whom 1,691 were included in the analysis (Supplementary Figure 1). Of these patients, 216 (12.8%) were aged 18 to 49 years, with 79.6% being male, while 1,475 patients were aged 50 years or older, accounting for 87.2% of the study population, of which 64.6% were male subjects.

The younger patients in the study had a lower median [interquartile range, IQR] baseline NIHSS score compared to the older patients (14 [9–19] vs. 17 [12–21], *P* < 0.001), and also had a lower baseline systolic blood pressure (140 vs. 146 mmHg, *P* = 0.007) (Table 1). The prevalence of (cardiovascular) disease including hypertension, diabetes, coronary artery disease, and atrial fibrillation, was lower in the younger patient group. No differences were observed between the groups in terms of prior use of antiplatelet agents, anticoagulants, or intraprocedural use of heparin and GPIIb/IIIa inhibitor IVT (all *P* > 0.05). Although baseline process measures, such as door-to-puncture time in minutes, were similar between the two groups, the onset-to-puncture time was longer in the younger patients (357 vs. 294, *P* = 0.001).

**Table 1:** Baseline characteristics of patients undergoing thrombectomy with Age <50 y versus Age ≥50 y.

**Baseline and procedure variables**	**Age <50 y (*n* = 216)**	**Age ≥50 y (*n* = 1475)**	***p*-value**
Male sex	172 (79.6)	953 (64.6)	< 0.001
History of hypertension	77 (35.7)	890 (60.3)	< 0.001
History of diabetes mellitus	23 (10.7)	284 (19.3)	< 0.001
History of dyslipidemia	15 (6.9)	139 (9.4)	0.237
History of coronary heart disease	11 (5.1)	244 (16.5)	< 0.001
History of atrial fibrillation	24 (11.1)	502 (34.0)	< 0.001
History of stroke	35 (16.2)	346 (23.5)	0.017
Cigarette smoking			< 0.001
Never smoker	105 (48.6)	897 (60.8)	
Ex-smoker	102 (47.2)	459 (31.1)	
Current smoker	9 (4.2)	119 (8.1)	
Systolic blood pressure, median (IQR), mmHg	140 (128–160)	146 (132–161)	0.007
NIHSS score, median (IQR)	14 (9–19)	17 (12–21)	< 0.001
ASPECTS, median (IQR)^a^	9 (7–10)	10 (7–10)	0.394
Occlusion circulation			0.086
Anterior circulation occlusion	159 (73.6)	1162 (78.8)	
Posterior circulation occlusion	57 (26.4)	313 (21.2)	
Occlusion site			0.558
Internal carotid artery	47 (21.8)	381 (25.8)	
Middle cerebral artery M1 segment	95 (44.0)	642 (43.5)	
Vertebro-basilar artery	1 (0.5)	5 (0.3)	
Other intracranial arteries	73 (33.8)	447 (30.3)	
Underlying ICAD	85 (39.4)	423 (28.7)	0.001
Stroke subtype by TOAST criteria			< 0.001
Large artery atherosclerosis	110 (50.9)	744 (50.4)	
Cardioembolism	43 (19.9)	521 (35.3)	
Other or unknown etiology	61 (28.2)	135 (9.2)	
Prior use of antiplatelet agents	31 (14.4)	251 (17.0)	0.326
Prior use of anticoagulants	13 (6.0)	56 (3.8)	0.123
Type of anesthesia			0.434
Local anesthesia only	98 (45.4)	634 (43.0)	
Local anesthesia plus sedation	89 (41.2)	592 (40.1)	
General anesthesia	29 (13.4)	249 (16.9)	
Stentriever-first techniques	192 (88.9)	1287 (87.3)	0.498
Aspiration-first	39 (18.06)	303 (20.54)	0.395
Intra-arterial thrombolysis	23 (10.7)	121 (8.2)	0.229
Balloon angioplasty	54 (25.0)	322 (21.8)	0.296
Stenting	45 (20.8)	272 (18.4)	0.400
Intra-procedural use of heparin	115 (53.2)	722 (49.9)	0.239
Intra-procedural use of GPIIb/IIIa	120 (55.6)	759 (51.5)	0.260
Door-to-puncture time, median (IQR), min	121 (78–182)	120 (80–179)	0.625
Onset-to-puncture time, median (IQR), min	357 (230–490)	294 (210–434)	0.001

Values are numbers with percentages in parentheses, unless indicated otherwise.

a ASPECTS for anterior circulation stroke; and pc-ASPECTS for posterior circulation stroke. ASPECTS, Alberta stroke program early CT score; ICAD, intracranial atherosclerotic disease; IQR, interquartile range; NIHSS, National Institutes of Health Stroke Scale; pc-ASPECTS, posterior circulation Alberta stroke program early CT score; TOAST, Trial of ORG 10172 in Acute Stroke Treatment. Ex-smoker is defined as an individual who has a prior history of tobacco use, but has presently abstained from smoking.

Younger patients had a lower prevalence of cardioembolism stroke etiology by TOAST criteria (19.9 vs. 35.3% in older patients) and a higher prevalence of stroke etiology classified as other or unknown (28.2 vs. 9.2% in older patients). The prevalence of large-artery atherosclerosis stroke etiology was similar between the two age groups (50.9 vs. 50.4%). Strikingly, younger patients had a higher prevalence of symptomatic underlying ICAD compared to older patients (39.4 vs. 28.7%, *P* = 0.001).

**Figure 1:**
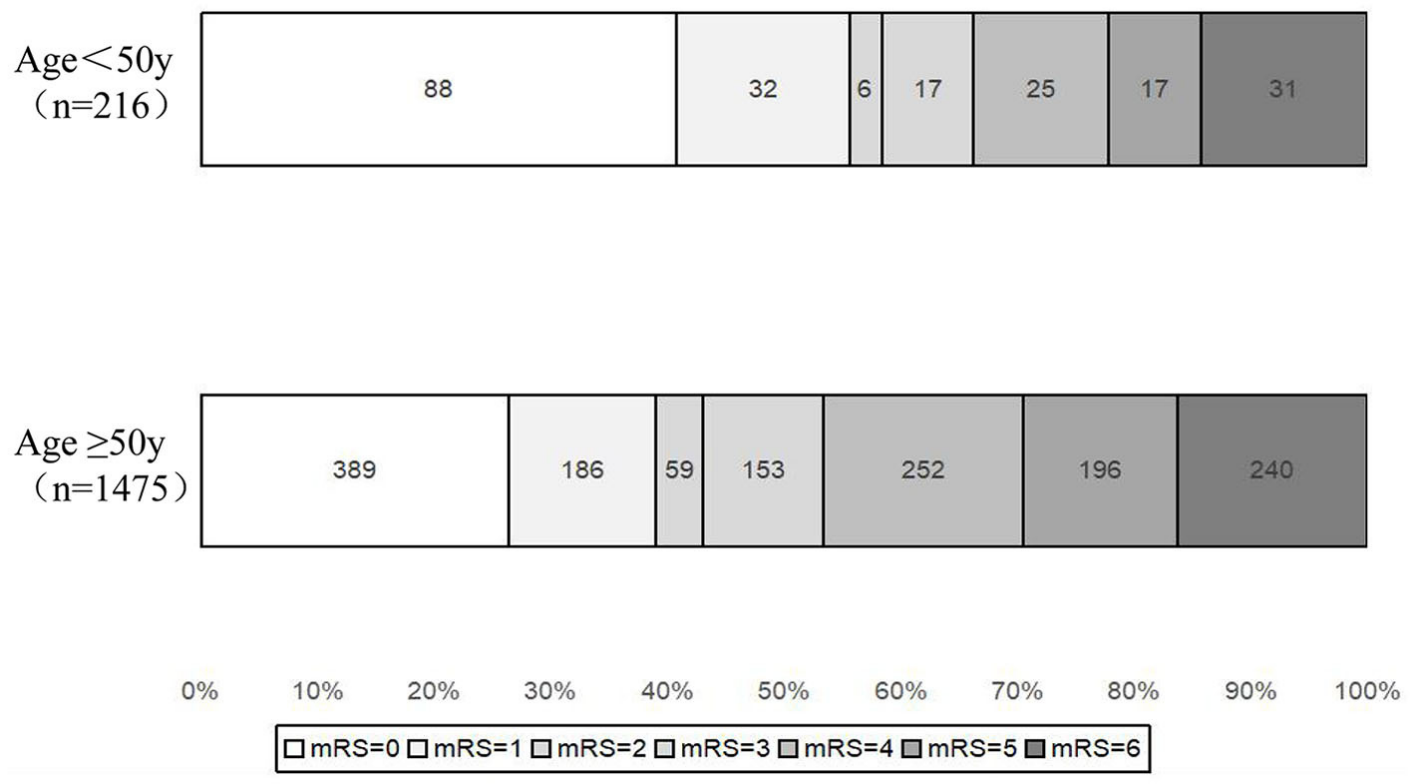
Shift on mTICI and 90-day mRS score stratified by age <50 and ≥50 years old in the post-matched population. mRS, modified Rankin scale.

Table 2 shows the clinical outcomes and treatment times. After the adjustment of covariates, older patients had a less favorable outcome compared to younger patients, with an adjusted odds ratio (aOR) of 0.76 (95% CI, 0.58–0.99), as shown in Figure 1. Older patients also had a lower frequency of functional independence (mRS scores 0–2) than younger patients, with an aOR of 0.7 (95% CI, 0.51–0.97), as well as an excellent outcome (mRS scores 0–1) with an aOR of 0.67 (95% CI, 0.49–0.92). However, there was no significant difference in favorable outcomes (mRS scores 0–3) between the two groups, with an aOR of 0.79 (95% CI, 0.57–1.10). No significant differences were observed in the number of MT attempts, puncture-to-recanalization time, successful recanalization rate, mortality, change in NIHSS score at 24 h and 7 days, any ICH, sICH, incidence of preoperative distal embolism or embolism to new territory, and intraoperative complications (all *P* > 0.05) between the young and older patients.

**Table 2:** Outcome measures of patients undergoing thrombectomy with Age <50 y versus Age ≥50 y.

**Outcome variables**	**Age <50 y**	**Age ≥50 y**	**Unadjusted analysis**		**Adjusted model 1a**		**Adjusted model 2b**	
			**Effect size (95% CI)**	***p*** **-value**	**Effect size (95% CI)**	***p*** **-value**	**Effect size (95% CI)**	***p*** **-value**
**Primary outcome**								
mRS at 90 d, median (IQR)	1 (0–4)	3 (0–5)	0.57 (0.44–0.74)^c^	< 0.001	0.76 (0.58–0.99)^c^	0.045	0.76 (0.58–1.00)^c^	0.049
**Secondary outcomes**								
mRS 0–1 at 90 days	120 (55.6)	575 (39.0)	0.51 (0.38–0.68)^d^	< 0.001	0.67 (0.49–0.92)^d^	0.013	0.68 (0.50–0.93)^d^	0.014
mRS 0–2 at 90 days	126 (58.3)	634 (43.0)	0.54 (0.40–0.72)^d^	< 0.001	0.70 (0.51–0.97)^d^	0.029	0.71 (0.52–0.96)^d^	0.027
mRS 0–3 at 90 days	143 (66.2)	787 (53.4)	0.58 (0.43–0.79)^d^	< 0.001	0.79 (0.57–1.10)^d^	0.155	0.80 (0.58–1.10)^d^	0.160
Death within 90 days	31 (14.4)	240 (16.3)	1.16 (0.77–1.74)^d^	0.473	0.71 (0.45–1.12)^d^	0.140	0.71 (0.46–1.09)^d^	0.120
Change in NIHSS score at 24 h, median (IQR)^e^	5 (0–9)	5 (0–9)	0.44 (-0.88–1.75)	0.515	−0.53 (-1.78–0.72)	0.404	−0.42 (−1.81–0.96)	0.550
Change in NIHSS score at 7 days, median (IQR)^f^	7 (3–11)	8 (3–13)	1.32 (-0.10–2.74)	0.068	−0.15 (-1.50–1.21)	0.833	−0.00 (−1.49–1.48)	0.997
Puncture to recanalization/end of procedure time (IQR)	76 (50–135)	86 (53–127)	2.39 (−6.26–11.05)^e^	0.588	−2.50 (−11.29–6.28)^e^	0.576	−2.10 (−11.24–7.03)^e^	0.652
MT PASSES (*n*, %)	1 (1–2)	1 (1–2)	−0.09 (-0.31–0.12)	0.393	−0.00 (−0.22–0.22)	0.995	0.02 (−0.21–0.25)	0.865
Successful recanalization at final angiogram^f^	197 (91.2)	1312 (89.0)	0.78 (0.47–1.28)	0.319	0.75 (0.44–1.28)	0.298	0.80 (0.48–1.35)	0.407
**Safety outcomes**								
Any ICH within 24 hours^g^	36 (17.0)	326 (22.9)	1.45 (0.99–2.12)	0.054	1.21 (0.81–1.81)	0.348	1.20 (0.81–1.79)	0.363
Symptomatic ICH within 24 hours ^g^	11 (5.2)	103 (7.3)	1.42 (0.75–2.70)	0.279	1.07 (0.54–2.09)	0.850	1.02 (0.53–1.99)	0.948
Incidence of intraoperative distal embolism or ectopic embolism	7 (3.2)	78 (5.3)	1.67 (0.76–3.66)	0.203	1.36 (0.60–3.09)	0.469	1.40 (0.62–3.17)	0.415
Intraoperative complications	192 (88.9)	1271 (86.2)	0.78 (0.50–1.22)	0.276	0.87 (0.54–1.40)	0.563	0.89 (0.55–1.42)	0.614

Data are shown as the event number/total number (%), unless otherwise indicated. ^a^Adjusted for the baseline variables with a significant difference of *p* < 0.05 between both groups, including Male sex, History of coronary heart disease, History of atrial fibrillation, History of stroke, Cigarette smoking, NIHSS score, Underlying ICAD, Stroke subtype by TOAST criteria, Balloon angioplasty, Stenting, Intra–procedural use of GPIIb/IIIa, Onset–to–puncture time. ^b^Adjusted for the propensity score. ^c^The common OR values were calculated using an ordinal logistic regression model and indicated the odds of improvement of 1 point on the mRS at 90 days. ^d^The OR values were calculated using a binary logistic regression model. ^e^The β-coefficients were calculated using a generalized linear model. ^f^Defined as mTICI of 2b−3. ^g^According to the Heidelberg Bleeding Classification. CI, confidence interval; ICH, intracranial hemorrhage; IQR, interquartile range; mRS, modified Rankin Scale; mTICI, modified Thrombolysis in Cerebral Infarction; OR, odds ratio; TOAST, Trial of ORG 10172 in Acute Stroke Treatment.

**Figure 2:**
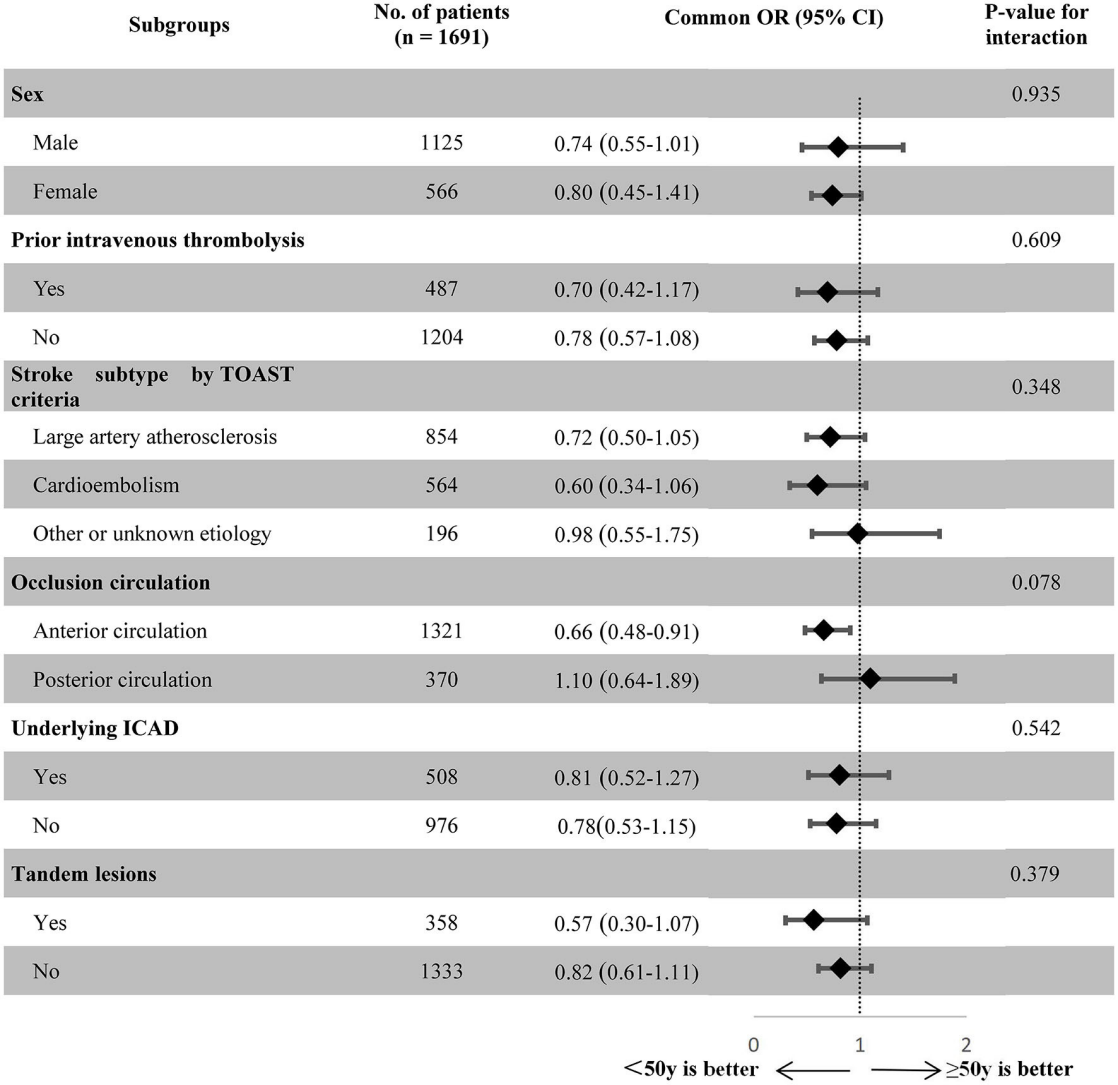
Treatment modification on the 90-day mRS score ordinal distribution according to exploratory subgroups in the post-matched population. TOAST, Trial of ORG 10172 in Acute Stroke Treatment; ICAD, intracranial atherosclerotic disease; OR, odds ratio; CI, confidence interval.

The exploratory subgroup analyses demonstrated that there was no significant heterogeneity in the treatment effects on the primary outcome across the subgroups, as stratified by sex, prior intravenous thrombolysis, stroke subtype according to TOAST criteria, occlusion circulation, symptomatic underlying ICAD, and tandem lesions (all *p*-values for interactions were >0.05), as shown in Figure 2.

## Discussion

The objective of this study was to evaluate the clinical outcomes following EVT and to determine the stroke characteristics of young patients undergoing EVT for stroke treatment. Our findings revealed that, despite presenting with a longer onset to puncture time, younger patients demonstrated a superior clinical outcome than their older counterparts. Furthermore, we noted a lower occurrence of cardioembolic stroke etiology among younger patients as compared to older patients. The prevalence of large-artery atherosclerosis stroke etiology was comparable in both age groups. However, younger patients exhibited a greater incidence of symptomatic underlying ICAD as compared to their older counterparts.

The ANGEL-ACT Registry revealed that approximately 13% of patients who underwent EVT for stroke treatment were young individuals. This percentage is marginally higher than the data on young patients treated with EVT reported by other studies (15–17). At baseline, prior smoking habits were more prevalent among the younger group (47.2%) compared to the older group, which likely explains the higher prevalence of ICAD in the young population. However, hypertension (60.3%), diabetes mellitus (19.3%), coronary heart disease (16.5%), atrial fibrillation (34%), and prior stroke (23.5%) were significantly more frequent in the older group. These observations suggest that lifestyle-related risk factors may be the most prevalent risk factors for stroke in young adults, rather than pre-existing disease (18–20). Our study confirms this finding and highlights it as a global health issue rather than an issue specific to certain countries. This is of public health significance as stroke in younger patients can have a more severe and long-lasting impact on their ability to work and participate in daily activities (4, 5). Survivors of stroke may experience disability, with a reported 60% of survivors being affected (21). Returning to society immediately after stroke can also be challenging (22). The increasing incidence of stroke in young adults is anticipated to have significant personal as well as social costs. This may also highlight the need for increased awareness and education on stroke prevention in the young population.

Interestingly, although there was no significant difference observed in door-to-puncture time and puncture-to-recanalization time, we noted a considerably longer time from onset to puncture time in the younger patient group compared to the older patient group. This may suggest a prehospital delay in young patients with stroke. Our findings highlight the notable improvement in stroke management workflow in China. However, the prehospital delay requires further attention from the government and community efforts to improve stroke education in the young population. Previous studies have reported that a shorter duration from onset to arrival is a predictor of favorable clinical outcomes and an overall survival rate (23). This delay may also explain the relatively higher mortality rate at 90 days in our study (14.4%) compared to other studies.

Our study's finding that young patients with stroke have a high rate of favorable outcomes aligns with previous reports (8, 9, 24, 25). Our observation regarding mortality and sICH is consistent with Shi et al. study (26), which found no significant difference between young patients with stroke and those above 50 years who received intravenous thrombolysis (IVT). A plausible explanation for the better clinical outcomes observed in young patients with stroke may be their overall better health and fewer comorbidities. Another explanation could be the availability of more comprehensive rehabilitation options for young patients.

It is noteworthy that in our cohort, large-artery atherosclerosis was the most prevalent etiology of stroke in young patients (50.9%), followed by unknown or other causes (28.2%) and cardioembolism (19.9%). Our findings align with a large-scale study of Chinese ischemic stroke patients aged 15–49 years, (27) which reported that large-artery atherosclerosis accounted for 43.7% of total ischemic strokes. This is in contrast to studies conducted in Western countries (Supplementary Table 1), such as the MR CLEAN Registry (15), which found cardiogenic disease (14.8%) and cervical arterial dissection (16.2%) as common causes, followed by large-artery atherosclerosis (10.0%). The European-Asian cohort study reported that arterial dissection (21.5%) was the most frequent cause. In the GSR-ET study (17), cardiogenic diseases (28.3%) and unknown etiologies (30.1%) were the primary causes. Understanding the etiology of ischemic stroke in young adults in various populations is critical to grasping the stroke characteristics in different populations and developing effective mechanical thrombectomy strategies.

The current study exhibits limitations that must be acknowledged. First, despite adjustment and propensity matching techniques, the non-randomized study design may result in measurable or immeasurable variables that are not mentioned in this study, which could act as confounding factors. Moreover, the absence of a control group in the ANGEL-ACT registry precludes the inclusion of data on stroke patients who did not undergo EVT, thereby rendering a comparison of outcomes between EVT and non-EVT patients unfeasible. Second, even though all patients were treated in accordance with the recommended guidelines, there were differences in endovascular approaches and postoperative management in each center, which could impact the outcomes of the study. Nonetheless, the strength of the study lies in the fact that a large number of young stroke patients were included in the present study. We did not examine other potential risk factors that may affect stroke etiology in the young population, such as arterial dissection, substance abuse, and genetic predisposition to a hypercoagulable state. As our study was conducted in a Chinese population, our findings may not be generalizable to patients of other ethnicities. It is expected that the findings of this study will aid in tailoring the additional workup for young patients treated with EVT and will facilitate the counseling of these patients and their relatives regarding expected outcomes.

## Conclusion

In the present study, our findings revealed that a notable proportion of stroke patients (approximately 13%) who underwent EVT were <50 years of age. The incidence of symptomatic underlying ICAD was observed to be comparatively higher in this subset of younger patients. Despite the presence of prehospital delays during the treatment course, the study demonstrated that younger patients were more likely to achieve recovery toward independent living following EVT.

## Data availability statement

The original contributions presented in the study are included in the article/Supplementary material, further inquiries can be directed to the corresponding author.

## Ethics statement

The studies involving humans were approved by the Ethics Committees of Beijing Tiantan Hospital and each participating site. The studies were conducted in accordance with the local legislation and institutional requirements. The participants provided their written informed consent to participate in this study.

## Author contributions

BH: Conceptualization, Data curation, Formal analysis, Methodology, Writing—original draft. R: Conceptualization, Data curation, Formal analysis, Investigation, Writing—original draft. DS: Formal analysis, Investigation, Writing—original draft. XT: Formal analysis, Writing—review and editing. BJ: Formal analysis, writing—review and editing. AW: Methodology, Writing—review and editing. DM: Supervision, Writing—review and editing. FG: Supervision, Writing—review and editing. NM: Supervision, Writing—review and editing. TN: Conceptualization, Supervision, Validation, Writing—review and editing. ZM: Conceptualization, Supervision, Validation, Writing—review and editing.

## References

[1] Goyal M Menon BK van Zwam WH Dippel DW Mitchell PJ Demchuk AM. Endovascular thrombectomy after large-vessel ischaemic stroke: A meta-analysis of individual patient data from five randomised trials. Lancet. (2016) 387:1723–31. 10.1016/S0140-6736(16)00163-X 26898852

[2] Berkhemer OA Fransen PS Beumer D van den Berg LA Lingsma HF Yoo AJ. A randomized trial of intraarterial treatment for acute ischemic stroke. N Engl J Med. (2015) 372:11–20. 10.1056/NEJMoa1411587 25517348

[3] Ekker MS Verhoeven JI Vaartjes I van Nieuwenhuizen KM Klijn CJM de Leeuw FE. Stroke incidence in young adults according to age, subtype, sex, and time trends. Neurology. (2019) 92:e2444–54. 10.1212/WNL.0000000000007533 31019103

[4] Kissela BM Khoury JC Alwell K Moomaw CJ Woo D Adeoye O. Age at stroke: Temporal trends in stroke incidence in a large, biracial population. Neurology. (2012) 79:1781–7. 10.1212/WNL.0b013e318270401d 23054237 PMC3475622

[5] Vestling M Tufvesson B Iwarsson S. Indicators for return to work after stroke and the importance of work for subjective well-being and life satisfaction. J Rehabil Med. (2003) 35:127–31. 10.1080/16501970310010475 12809195

[6] Chalouhi N Tjoumakaris S Starke RM Hasan D Sidhu N Singhal S. Endovascular stroke intervention in young patients with large vessel occlusions. Neurosurg Focus. (2014) 36:E6. 10.3171/2013.9.FOCUS13398 24380483

[7] Zanaty M Chalouhi N Starke RM Tjoumakaris S Hasan D Hann S. Endovascular stroke intervention in the very young. Clin Neurol Neurosurg. (2014) 127:15–8. 10.1016/j.clineuro.2014.09.022 25459237

[8] Yesilot Barlas N Putaala J Waje-Andreassen U Vassilopoulou S Nardi K Odier C. Etiology of first-ever ischaemic stroke in european young adults: The 15 cities young stroke study. Eur J Neurol. (2013) 20:1431–9. 10.1111/ene.12228 23837733

[9] Maaijwee NA Rutten-Jacobs LC Schaapsmeerders P van Dijk EJ de Leeuw FE. Ischaemic stroke in young adults: Risk factors and long-term consequences. Nat Rev Neurol. (2014) 10:315–25. 10.1038/nrneurol.2014.72 24776923

[10] Rutten-Jacobs LC Arntz RM Maaijwee NA Schoonderwaldt HC Dorresteijn LD van Dijk EJ. Cardiovascular disease is the main cause of long-term excess mortality after ischemic stroke in young adults. Hypertension. (2015) 65:670–5. 10.1161/HYPERTENSIONAHA.114.04895 25624336

[11] von Elm E Altman DG Egger M Pocock SJ Gotzsche PC Vandenbroucke JP. The strengthening the reporting of observational studies in epidemiology (strobe) statement: Guidelines for reporting observational studies. PLoS Med. (2007) 4:e296. 10.1371/journal.pmed.0040296 17941714 PMC2020495

[12] Jia B Ren Z Mokin M Burgin WS Bauer CT Fiehler J. Current status of endovascular treatment for acute large vessel occlusion in china: A real-world nationwide registry. Stroke. (2021) 52:1203–12. 10.1161/STROKEAHA.120.031869 33596674

[13] Wang Y Liu M Pu C. 2014 chinese guidelines for secondary prevention of ischemic stroke and transient ischemic attack. Int J Stroke. (2017) 12:302–20. 10.1177/1747493017694391 28381199

[14] Adams HP Bendixen BH Kappelle LJ Biller J Love BB Gordon DL. Classification of subtype of acute ischemic stroke. Definitions for use in a multicenter clinical trial. Toast. Trial of org 10172 in acute stroke treatment. Stroke. (1993) 24:35–41. 10.1161/01.STR.24.1.35 7678184

[15] Brouwer J Smaal JA Emmer BJ de Ridder IR van den Wijngaard IR de Leeuw FE. Endovascular thrombectomy in young patients with stroke: a MR clean registry study. Stroke. (2022) 53:34–42. 10.1161/STROKEAHA.120.034033 34872339

[16] Yeo LL Chen VHE Leow AS Meyer L Fiehler J Tu TM. Outcomes in young adults with acute ischemic stroke undergoing endovascular thrombectomy: a real-world multicenter experience. Eur J Neurol. (2021) 28:2736–44. 10.1111/ene.14899 33960072

[17] Weller JM Dorn F Meissner JN Stosser S Beckonert NM Nordsiek J. Endovascular thrombectomy in young patients with stroke. Int J Stroke. (2023) 18:453–61. 10.1177/17474930221119602 35912650

[18] Siriratnam P Godfrey A O'Connor E Pearce D Hu CC Low A. Prevalence and risk factors of ischaemic stroke in the young: a regional australian perspective. Intern Med J. (2020) 50:698–704. 10.1111/imj.14407 31211881

[19] Lasek-Bal A Kopyta I Warsz-Wianecka A Puz P Labuz-Roszak B Zareba K. Risk factor profile in patients with stroke at a young age. Neurol Res. (2018) 40:593–9. 10.1080/01616412.2018.1455367 29577820

[20] Bailey RR Phad A McGrath R Haire-Joshu D. Prevalence of five lifestyle risk factors among US Adults with and without stroke. Disabil Health J. (2019) 12:323–7. 10.1016/j.dhjo.2018.11.003 30448248 PMC6431268

[21] Pollock A St George B Fenton M Firkins L. Top 10 research priorities relating to life after stroke–consensus from stroke survivors, caregivers, and health professionals. Int J Stroke. (2014) 9:313–20. 10.1111/j.1747-4949.2012.00942.x 23227818

[22] Treger I Shames J Giaquinto S Ring H. Return to work in stroke patients. Disabil Rehabil. (2007) 29:1397–403. 10.1080/09638280701314923 17729085

[23] Szmygin M Sojka M Tarkowski P Pyra K Luchowski P Wojczal J. Predictors of favorable outcome after endovascular thrombectomy for acute ischemic stroke due to large vessel occlusion in young patients. Acta Radiol. (2022) 63:1689–94. 10.1177/02841851211056476 34766505

[24] Wiegers EJA Mulder M Jansen IGH Venema E Compagne KCJ Berkhemer OA. Clinical and imaging determinants of collateral status in patients with acute ischemic stroke in MR clean trial and registry. Stroke. (2020) 51:1493–502. 10.1161/STROKEAHA.119.027483 32279619

[25] Malik N Hou Q Vagal A Patrie J Xin W Michel P. Demographic and clinical predictors of leptomeningeal collaterals in stroke patients. J Stroke Cerebrovasc Dis. (2014) 23:2018–22. 10.1016/j.jstrokecerebrovasdis.2014.02.018 25088172

[26] Shi J Cao Y You S Huang Z Zhang X Liu H. Young stroke patients treated with intravenous thrombolysis have a more favorable outcome and mortality compared with older patients. Curr Neurovasc Res. (2017) 14:141–8. 10.2174/1567202614666170328095431 28356003

[27] Li F Yang L Yang R Xu W Chen FP Li N. Ischemic stroke in young adults of northern china: characteristics and risk factors for recurrence. Eur Neurol. (2017) 77:115–22. 10.1159/000455093 28052272 PMC5467437

