# Supplementary material for: Endovascular treatment for young patients with acute large vessel occlusion stroke in China: analysis of the ANGEL-ACT registry
Source: Front Neurol. 2023 Oct 20;14:1255043. doi: 10.3389/fneur.2023.1255043 (PMC10623312; doi:10.3389/fneur.2023.1255043)

Supplementary figure 1. Flow chart of patient selection.

Abbreviations: MT = mechanical thrombectomy.

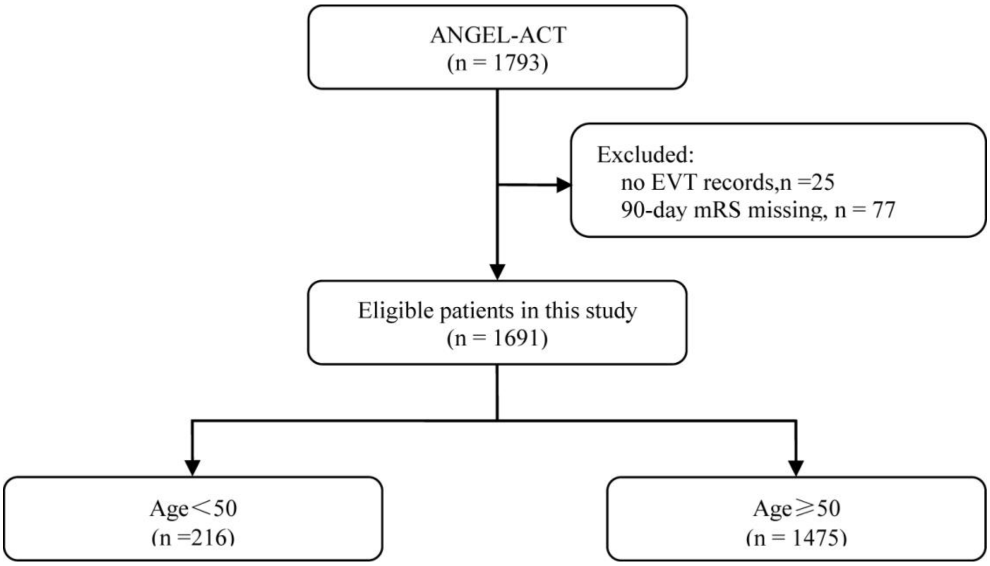

Supplement: Supplementary file 1 [file Image_1.pdf]
