# Supplementary material for: Endovascular treatment for young patients with acute large vessel occlusion stroke in China: analysis of the ANGEL-ACT registry
Source: Front Neurol. 2023 Oct 20;14:1255043. doi: 10.3389/fneur.2023.1255043 (PMC10623312; doi:10.3389/fneur.2023.1255043)
Supplement: Supplementary file 2 [file Table_1.pdf]

Supplementary table 1. Comparison between our cohort and other young patients cohorts

Characteristic	Our study (n = 216,12.8%)	GSR-ET (n = 363,5.5%)	MR CLEAN Registry (n = 310,9.5%)	European-Asian cohort (n = 275,5.3%)
Period	2017.11–2019.3	2015.7-2019.12	2014.3-2017.11	2014.8-2020.1
Region	China	German	Netherlands	Taiwan, Germany, Sweden and Singapore
Male sex, % (n)	172(79.6)	208(57.3)	145(47.0)	163(59.3)
History of hypertension	77(35.7)	111 (32.6)	46/307 (15.0)	103(37.5)
History of diabetes mellitus	23(10.7)	27 (7.9)	12/309 (3.9)	
History of dyslipidemia	15(6.9)	75 (22.0)	34/307 (11.1)	58(21.1)
History of coronary heart disease	11(5.1)		13/309 (4.2)	
History of atrial fibrillation	24(11.1)	20 (5.8)		26(9.5)
NIHSS score, median (IQR)	14(9-19)	13 (8–17)	14 (10 - 18)	14 (10-19)
Underlying ICAD	85(39.4)			
Current smoking	9(4.2)	124 (38.4)		
Stroke subtype by TOAST criteria				
Large artery atherosclerosis	110(50.9)	67 (19.5)	31 (10.0)	53(19.3)
Cardioembolism	43(19.9)	99 (28.9)	46 (14.8)	82(29.8%)
Other or unknown etiology	61(28.2)	197(51.6)	233(75.2)	140(50.9%)
Door-to-puncture time, median (IQR), min	121(78-182)	64 (44–96)	126 (50 - 214)	
Onset-to-puncture time, median (IQR), min	357(230-490)			220 (152-322)
Successful recanalization, n (%)	197(91.2)	314(89.0)	199 (64.2)	234(85.1%)
mRS 0–1 at 90-day, n (%)	120(55.6)	154 (53.3)		132 (48.0%)
mRS 0–2 at 90-day, n (%)	126(58.3)	205 (70.9)	189 (61.0)	182 (66.2%)
sICH within 24 h, n (%)	11(5.2)	10 (2.8)	9 (2.9)	18 (6.5%)
Mortality at 90-day, n (%)	31(14.4)	21 (7.3)	21 (6.8)	13 (4.7%)

GSR-ET：German Stroke Registry–Endovascular Treatment * Mean (standard deviation). -not given in the article. IQR interquartile range, NIHSS National Institute of Health Stroke Scale, IV intravenous,

GPI glycoprotein IIb/IIIa inhibitor, OPT onset to puncture time, PRT puncture to recanalization time, mRS modified Rankin Scale, sICH symptomatic

intracranial hemorrhage
